# The Effect of Lithium Salt with Ascorbic Acid on the Antioxidant Status and Productivity of Gestating Sows

**DOI:** 10.3390/ani12070915

**Published:** 2022-04-02

**Authors:** Konstantin Ostrenko, Roman Nekrasov, Anastasiya Ovcharova, Viktar Lemiasheuski, Ivan Kutin

**Affiliations:** 1 All-Russian Research Institute of Physiology, Biochemistry and Nutrition of Animals—Branch of the L.K. Ernst Federal Research Center for Animal Husbandry, VNIIFBiP, Borovsk 249013, Russia; naka7@yandex.ru (A.O.); lemeshonak@mail.ru (V.L.); kurookami@mail.ru (I.K.); 2 Skryabin, Academy of Veterinary Medicine and Biotechnology, Moscow 109472, Russia; 3 International Sakharov Environmental Institute, Belarusian State University, 220070 Minsk, Belarus; 4 L.K. Ernst Federal Research Center for Animal Husbandry, Podolsk 142132, Moscow Region, Russia; nek_roman@mail.ru

**Keywords:** sows, lithium ascorbate, antioxidant status, glutathione reduction system, oxidative stress, productivity

## Abstract

**Simple Summary:**

This research is aimed at studying the effect of lithium ascorbate on increasing the antioxidant status of gestational sows and reducing the level of lipid peroxidation products. The studies were conducted on gestational sows of the Irish Landrace breed. Thirty days after successful insemination, the sows of the experimental groups, E10, E5 and E2, began to receive lithium ascorbate powder together with compound feed in doses of 10, 5, and 2 mg/kg of live weight, respectively. Weighing and biochemical examination were carried out before the introduction of the substance, as well as on the 60th and 110th days of gestation. In the blood plasma of sows, the following were determined: malondialdehyde, reduced glutathione, oxidized glutathione, SH/SS ratio, activity of superoxide dismutase and glutathione peroxidase. The use of lithium ascorbate b caused a significant increase in the level of SH (reduced glutathione) by 21% (*p* < 0.05), a decrease in the level of SS (oxidized glutathione) by 17% (*p* < 0.05) and a decrease in the level of malondialdehyde by 60% (*p* < 0.05). The results obtained cause activation of the antioxidant defense system, reduce the risk of oxidative stress, and have a positive effect on the gestation process.

**Abstract:**

This research is aimed at the influence of different doses of lithium ascorbate on pigs’ diet estimation, at farrowing sows’ antioxidant status increase, and at lipid peroxidation product level decrease. The research was conducted in farrowing sows of the Irish landrace breed during the second farrow. Three groups of animals were formed, with ten livestock units in each. Thirty days after successful insemination, the sows of the E10, E5 and E2 experimental groups started receiving lithium ascorbate powder together with feed stuff in dosages of 10, 5 and 2 mg/kg of body weight, respectively. Their weighing and biochemical examinations were performed before the substance introduction as well as on the 60th and 110th days of pregnancy. The following were detected in sows’ blood plasma: malondialdehyde, reduced glutathione, oxidized glutathione, SH/SS ratio, superoxide dismutase and glutathione peroxidase activity. Lithium ascorbate usage during sows’ breeding cycle caused a significant increase in SH (reduced glutathione) level by 21% (*p* < 0.05), SS (oxidized glutathione) level decrease by 17% (*p* < 0.05), and malondialdehyde level decrease by 60% (*p* < 0.05). These data outline antioxidant defense system activization, reducing the risk of oxidative stress under the influence of feeding with lithium ascorbate. Lithium ascorbate in dosages of 10 mg/kg per body weight given together with feed stuff shows prominent adaptogene and stress protective features in the most effective way. The research conducted regarding lithium ascorbate usage for farrowing sows can reduce the negative consequences of oxidative stress, increase sows’ health preservation level, and contribute to fertility boost.

## 1. Introduction

Modern swine husbandry efficiency is closely connected with pig reproduction biotechnology. Valid methods ofanimal reproduction and breeding rate increase practical applications considerably, improving breeding stock exploitation together with securing production indices, high performance stability, and pork production technology costeffectiveness [1].

On the current level of physiological science development, special attention is given to the fundamental mechanisms of pig body life support identification and their usage in applied pig breeding [2]. It will allow for the development of new adaptive technologies of livestock reproduction and will update existing ones, correctly organizing pig herd reproduction technology based on consistent patterns of pig growth and development [3]. In present times, applied animal husbandry often refers to functional homeostasis regulation methods, of which one of them is new generation adaptogens usage, and of which lithium ascorbate falls under the same category.

The general nonspecific resistance of animals, which determines the adaptive capabilities of the organism and its ability to withstand stresses of any etiology, underlies animal health. The unified response of any organism to stress of any etiology is the formation of an excessive amount of overreactive free radicals. They are physiologically normal metabolites in stationary living conditions. The inability of the body to neutralize their increased formation is considered as the root cause of, if not all, the vast majority of pathological ailments. Stress, which entails a violation of physiological homeostasis, instantly and uncompromisingly initiates a unified set of responses of the body to overcome it. The response of any organism to any stress always begins with the instantaneous activation of the biosynthesis of catecholamines by the cerebral layer and corticoids by the adrenal cortex. They launch the most intricate complex characterized by an extensive network of catabolic reactions, including lipid peroxidation reactions, the products of which are super-reactive free radicals. The mechanism of action of Li+ is that it changes the level of K^+^ and Na^+^ in the blood and inside the cell. Li ions with the help of Na^+^, K^+^–ATPASE penetrate the cell membrane, enter the cell space, displace Na+ from the cell and prevent the entry of K^+^ into the cell. Thus, the nerve impulse is blocked, and the braking effect occurs. The use of lithium preparations increases the synthesis of neurotrophic factors and prevents the death of neurons through the modulation of the PI3K/Akt/GSK3 apoptosis cascade. Lithium accelerates the growth of neurons and increases their resistance to oxidative stress. Ascorbic acid is a natural antioxidant with proven effectiveness. Lithium ascorbate has a complex effect on neurohumoral status and normalizes it, while the effect of their use is a consequence of a decrease in the level of free radical oxidation, including lipoperoxidation.

Lithium ascorbate is a compound having both lithium normothymic activity and a vitamin effect of ascorbic acid [4,5,6]. Antioxidant properties of lithium salts in biological systems were evaluated by their outcome in oxidative stress marker presence, namely protein and lipid oxidative modification products in blood plasma after blood incubation with 85 mM (0.5%) alcohol in vitro. The final concentration of all compounds in the tests comprised 1.2 mmol/L equivalent to lithium ions, given that the concentration corresponds to the therapeutic lithium concentration in patients’ blood during mental diseases treatment, which allows us to extrapolate lithium effects found in vitro to their effects in vivo [7]. As it is known that lithium salts also have an expressed systematic effect on affection and are able to normalize it, the ability of lithium salts to protect biomolecules from oxidative damage found as a result of the present research allows us to provide evidence regarding the possibility of these compounds’ usage in the intensive industrial pig-breeding cycle. It is obvious that attraction–defensive effect mechanisms are not limited by their direct ability to neutralize free radicals [8,9].

Increases in stress resistance and efficiency will definitely have a positive impact on the glutathione reduction system, with joint processes of free radical performance decreasing in animal bodies, causing optimization in neuro-metabolic processes [10]. The given parameters can be seen as lithium ascorbate usage efficiency markers with sows, especially during the breeding cycle.

It is known that sub-effective doses of lithium chloride (10 mg/kg) combined with a sub-effective dose of ascorbic acid (0.1 mg/kg) produce a synergistic antidepressant-like effect [11].

In the study of reproductive toxicity in rats, lithium carbonate doses of 5, 15 and 45 mg/kg body weight/day (0.95, 2.9 and 8.6 mg Li^+^/kg body weight/day) were studied. Toxic effects of lithium carbonate were not seen in the reproductive and developmental organs [12].

Lithium in the form of carbonate and partially in the form of chloride and hypochlorite is being comprehensively investigated in relation to its reproduction/fertility and its developmental toxicity profile. Studies on rats, as well as exploratory screening studies, mainly concerning intrauterine toxicity, are available on rats, rabbits, mice, monkeys and pigs. As for lithium ascorbate, not enough studies have been conducted on pigs yet. The invention (United States patent application ser. no. 15/950,194, filed on 11 April 2018) discovers the possibility of using lithium ascorbate as an agent with antistress, anxiolytic and antidepressant activity.

Previously, we conducted a study on the effect of lithium ascorbate in fattening pigs in dosages of 2, 5, 10 mg/kg of live body weight. In the course of studies, a dose-dependent positive dynamic was found to increase the productive growth in pigs and reduce the risk of developing PSS (porcine stress syndrome) during pig slaughter [13].

The aim of this research is to study the different dosages of adaptogene and antioxidant lithium ascorbate and their influence onpigs’ diet and on farrowing sows’ antioxidant status and farrow indices. Similar research has never been conducted before.

## 2. Materials and Methods

### 2.1. Supplement Used in the Study

Lithium ascorbate synthesis (C6H7O6Li) was conducted at the All-Russian Research Institute of Physiology, Biochemistry and Nutrition of Animals, in the immunologic engineering and microbiology laboratory. Lithium ascorbate at 77.42% yield was received through the exposure of crystallized lithium hydroxide (0.0305 mole) on homogenous 20% ascorbic acid aqua solution (0.0284 mole), subsequently with pH reaction 7.5. Further, the solution received was evaporated in a high vacuum rotary evaporator (MID R205B, LLC MIDA, Moscow, Russia) and dried at a temperature of 45 °C for 6 h (hot-air sterilizer IKAOven 125 basic dry, IKA^®^, Guangzhou, China). The powder received had no smell and contained 3.18% of lithium.

### 2.2. Animals

This research was conducted at the swine farming enterprise “Rodina” in the Maloyaroslavets district of Kaluga region, the Russian Federation. For the research, we took 40 sows of Irish landrace breed during the second farrow. The sample of animals used in the experiment was carried out by their custom herd of the farm in the amount of 120 heads. The animals were selected taking into account the main criteria: age, farrowing, weight, physical condition, and confirmed fertilization. Confirmation of fertilization was performed using ultrasound scanner for pigs MULTISCAN M1 (Multiacan, Schippers, Holland) with a mechanical probe 14 days after re-insemination. In this regard, the study was planned from the 31st day of gestation of the sows, from the moment of transfer to the uterine machines. Four groups of farrowing sows were formed (3 experimentaland 1 control), with ten livestock units in each. The animals were chosen taking into consideration their weight and reproductive indices of the first farrow. Thirty days after successful insemination, the pigs from the experimental groups were given powdered lithium ascorbate in different dosages, together with the main ration for a long time on a daily basis. Controlgroup (C) had only the main ration, E10 group (E10) had main ration +10 mg of lithium ascorbate per 1 kg of body weight, E5 group (E5)had basic main ration +5 mg of lithium ascorbate per 1 kg of body weight, E2 group (E2) hadmain ration +2 mg of lithium ascorbate per 1 kg of body weight. They were fed lithium ascorbate as part of the feed for which the experimental batches were prepared, and the animals always had free access to fresh water.

The animalsofthe experimental and control groups were kept on the same premises in individual pens, after the groups were organized, for exact main rationand lithium ascorbatedosing.

Apart from that, the animals’diet and technological processes were the same and met all the requirements of pig feeding and keeping (Table 1).

### 2.3. Sows’ Weight and Fertility Indices

The sows were weighed on the 30th, 60th, 90th and 110th days of gestation. Weighing was performed on platform weigher model 4D-PM-10/10_A up to 500 kg (МАСCА-К, Saint Petersburg, Russia).

When the gestation period was over, the main criteria of farrow were registered, namely the number of piglets, litter weight, and number of alive and stillborn piglets.

Litter weight was determined by group weighing of all newborn piglets, and later, the average weight of a piglet was defined on weighing scales MK_АВ20 (МАСА-К, Saint Petersburg, Russia) [14]. The pen for farrowing sows was suitable for a gestational sow with weight parameters of 150–300 kg. The width of the machines was from 50 to 70 cm and length was changeable from 140 cm and more, depending on the length of the pig. The height was up to 110 cm, and the distance from the floor to the bottom pipe was 30–33 cm.

Conditions of detention: air temperature +16–25 °C; relative humidity 45–70%.

### 2.4. Blood Samples Analysis

On the 60th and 110th days of gestation, blood samples from sows’ jugular vein were collected before the morning feed. The samples were placed into vacuum test tubes with heparin (MiniMed, 4 mL, 13 × 100 mL, Bryans, Russia). During every examination, the concentration of the following was determined in blood plasma: malondialdehyde (nm/mL), reduced glutathione (umol/L), oxidized glutathione (umol/L), SH/SS ratio level, superoxide dismutase (Units) and glutathione peroxidase (Units) activity [15].

Examination was performed straight after samples collection during 12 h. Blood samples were centrifuged with 3200× *g* for 10 min in centrifuge (Armed LC-04B, Armed, Moscow, Russia). Samples examination was conducted in the immunologic engineering and microbiology laboratory of the All-Russian Research Institute of Physiology, Biochemistry and Nutrition of Animals—branch of the L.K. Ernst Federal Research Center for Animal Husbandry.

#### 2.4.1. Glutathione Chromatographic Determination

Reduced glutathione (GSH) reacts with ortho-phthalaldehydeand creates a stable and strongly fluorometric tricyclic derivative with pH 8, while oxidized glutathione (GSSG) reacts with ortho-phthalaldehyde with pH 12. While the GSSG level was being measured in the blood plasma, GSH was mixed with *N*-ethylmaleimide. Reverse phase columns were used for separation, namely Discovery C18 (Sigma-Aldrich, Meguro, Tokyo, Japan), 150 × 4 mm, 5 micron. A mixture of methanol and 25 mmol of sodium hydrogen phosphate (15:85), pH = 6.0, was used in the mobile phase. This method of analytical performance was satisfactory for GSH and GSSG. Within the analysis limits, the coefficients were 4.3% and 5.2% for plasma. Between the tests, variations of the coefficients were 6.9% and 7.8% for plasma. The outcome was distributed as follows: 94.1% (7.5%) and 103.5 (8.5%) for plasma. The calibration plot was linear within the whole range studied [16].

#### 2.4.2. Determination of the Activity of Superoxide Dismutase

Superoxide dismutase (SOD) activities were measured using standardized kits (Cayman Chemical) according to the manufacturer’s instructions. The absorbance was measured using a Tecan microplate reader (Tecan, SunRise, Männedorf, Austria). SOD activity was measured at 440–460 nm, and the results were expressed as u/mL. The assay allows for the measurement of all three types of SOD enzymes and uses tetrazolium salt for detection of superoxide radicals generated by hypoxanthine and xanthine oxidase. One unit was defined as the amount of enzyme needed to exhibit 50% dismutation of the superoxide radical. Superoxide dismutase activity is standardized using the cytochrome c and xanthine oxidase coupled assay. The SOD assay kit intra-assay CV was 3.2%, and the inter-assay CV was 3.7%. We obtained an intra-assay CV of 9.31% and an inter-assay CV of 17.08% [16,17].

#### 2.4.3. Malondialdehyde Determination Technique

Malondialdehyde (MDA) is one of the free radical lipid peroxidation products. Its accumulation shows the oxidative stress level in a body. A well-known method of MDA level measurement is its reaction with thiobarbituric acid, when as a result, pink chromogen is created, called trimethine complex. The MDA concentration level was determined according to this chromogen transmission optical density level, received in spectrophotometer, where the research subject was blood plasma or blood serum. The quality of the trimethine complex created corresponds with the reacted MDA quantity; therefore, its concentration can be calculated when the optical density of the sample under analysis is known. This method of oxidative stress level determination according to MDA issensitive. Even a small amount of sample (less than 0.1 mL) will determine the substance concentration level quite precisely [18].

#### 2.4.4. Glutathione Peroxidase Determination Technique

Glutathione peroxidase (GPO) reconstructs H_2_O_2_ and organic ROOH, respectively, up to water or up to alcohol. GPO neutralizes lipid peroxides such as linoleic and octadecatrienoic acid hydroperoxides, cholesterol 7P hydroperoxide and some other synthetic materials. GPO neutralizes peroxynitriteas well: 2GSH + ONOO ⟶ G-S-S-G + H_2_O + NO_2_. The highest GPO activity is observed in the liver, red blood cells, and suprarenal glands, as well as in average activity in the lungs and heart, and lower activity in muscles. A deficiency or inhibition of GPO leads to lipid peroxidation. Enzyme activity depends onthe number of peroxides formed. Enzymes are resistant to cyanide and azide activity in the case of the presence of reduced glutathione GSH. The test principle consists of the following: glutathione peroxidase (GPO) catalyzes the reaction of glutathione (GSH) interaction with tert-buty l hydroperoxide (TBH, pilot substrate).

GSH + TBHP = (GPx) = GSSO + reduced TBHP.

Enzyme activity is estimated according to GSH content change in the samples before and after incubation, with a pilot substrate during the color test.

The calculation is:

$$A=\frac{AD\times V\times 1000}{V_{np}\times s\times \mathrm{protein}\times d}$$

*AD* is the difference between the experimental and control samples’ transmission optical densities; *V* is the volume of the supernatant, used for GPO concentration determination (0.1 mL); *d* is the length of the experimental dish optical path (1 cm); *V* is the volume of the reaction mixture (2.775 mL); 1000/protein is the coefficient of remeasurement for g/protein [19].

### 2.5. Statistical Analysis

Statistical analysis was carried out by least squares mean comparisons using PDIFF option of the general linear model procedure (SAS, 2002; SAS Inst. Inc., Cary, NC, USA). The results are presented as mean values and standard deviations. The results were analyzed using the two-way analysis of variance (ANOVA) considering two factors: evaluation of the results taking into account the period of gestation and comparison with the control group. Each group was considered as an experimental unit of measurement of the parameters of BW and reproductive parameters, with each animals an experimental unit for the analysis of blood parameters.

Statistical 7.0 processing of the data obtained was carried out using Statistics for Windows software, version 6.0 (Microsoft Corporation, Redmond, WA, USA). Statistical differences were considered highly significant at *p* < 0.01 and significant at *p* < 0.05.

## 3. Results

### 3.1. Reproductive Indicators of Sows

Sow body weight changed with a certain dependance on lithium ascorbate content in feed stuff. The highest level of weight gain was found in the sows of the E10 and E5 experimental groups, which exceeded the results of the control group by 5.6% and 4.3%. In addition, in the E10 and E5 groups, the highest body weight of the piglets born was found (Table 2). Lithium ascorbate in addition to the feed stuff contributed to the sow fertility level increase in the E10 group by 37%, in the E5 group by 30%, in the E2 group by 13% in comparison to the animals in the control group. In the E10 and E5 groups, there were no stillborn piglets; in the E2 group, their number was less in comparison with the control group. All born piglets were healthy with a normal weight. Litter weight in the experimental group was higher than in the control one (Table 2 and Table 3).

### 3.2. Indicators Antioxidant Status of Sows

The SH/SS ratio level with the sows from the experimental groups was higher than the corresponding level of the SH/SS ratio in the control group on the 60th and 110th daysof gestation, and this effect size decreased as the substance dose was being reduced (Table 4).

Regarding the set of metrics describing the glutathione reduction system condition, the following changes can be marked in the sows’ blood of the experimental group (Table 4).

There was no proven difference in reduced glutathione concentration increase. A decrease in the oxidized glutathione level was stated in the E10 group by 17.5% (*p* < 0.05).

In the other groups, there was a tendency to increase SS indices when the lithium ascorbate dose was decreased. Proven changes were found in the SH/SS ratio. The E10 group difference between the experimental and control groups was higher by 43% (*p* < 0.05); in the E5 group, it was higher by 1.33% (*p* < 0.05). Malondialdehyde concentration was reduced by 38% in the E10 group (*p* < 0.01) and by 30% in the E5 group (*p* < 0.05). In the E10 group, the glutathione peroxidase level was higher by 12.6%, and in the E5 group by 9%. Superoxide dismutase activity was higher in the E10 group by 19.4% (*p* < 0.05).

## 4. Discussion

The usage of antioxidants in pigs’ diet for different gender and different age groups has a positive effect on the animal’s health and the products received from them [20,21,22,23]. The following can be used as antioxidants in different animal and bird diets: green tea powder [24], resveratrol [25], and cucurmin [26]. These compounds have high antioxidant activity, which corresponds with our research in antioxidant studies, in particular in lithium ascorbate, especially during the period when sows are the most sensitive to oxidative stress. In the research of Palade et al. [21], it was shown that the addition of hemp seeds to sow diet during the latest periods of gestation has a positive effect on the oxidative status both of sows and of blood at the expense of antioxidant enzyme activity increases and general antioxidant capacity. In the research, the quoted SOD and GPX main indicator levels increased in connection with the addition of linseed. Lithium ascorbate usage also has a prominent antioxidant effect [20].

Studying these indices, we based ourselves on facts and circumstances that the reductive–oxidative balance in a body is determined by donor–acceptor relationships. Eventually, reductive–oxidative metabolism regulation is determined by certain dynamic counterweighing among fundamental processes. This balance alteration within acceptable limits is used for multiple particular function regulation, but overstepping the limits will lead to pathology and to the death of a cell [10,27,28,29]. From this point of view, evaluation of the systems responsible for nonspecific body resistance and functional condition should be completed with an SH/SS system description, such as the thiol–disulfide ratio (TDR), i.e., the sulfhydryl and disulfide group number ratio plays an important part of the regulatory factor in reductive–oxidative metabolism.

Inorganic lithium salts have been used for more than 60 years and are used as medicines with a narrow therapeutic range in patients with bipolar disorders. Lithium carbonate is the most widely used lithium salt in medicine, followed by nitrate, acetate, sulfate and glutamate salts [30]. Low dose lithium chloride (10 mg/kg) in combination with a low dose of ascorbic acid (0.1 mg/kg) may have a synergistic effect [11]. In addition, based on the known data on chloride, carbonate, and other lithium salts, lithium ascorbate, as a less studied form, is certainly of interest. We have previously shown a significant neuroprotective effect of lithium ascorbate in the cultures of neurons subjected to glutamate stress, and the adaptogenic effect of this drug in various stress models in rats [4]. The experimental data confirm that when administered orally, under the action of lithium ascorbate, lithium ascorbate rapidly adapts the body to various stress factors in model studies during the first 7 days. The minimum dose of the drug (30 mg/kg) is not significantly inferior in effectiveness to higher doses (60–120 mg/kg).

In our research, we have determined linear dose dependability. Thus, an optimum dose that can activate antioxidant defenses is10 mg of lithium ascorbate per 1 kg of a sow’s body weight. In our opinion, the use of 10 mg of lithium was more effective, since in our work, the lowest doses were taken to study, which could be safe, first of all, for the body of pregnant sows and their offspring. The safety of the use of lithium components in radiation still raises questions. At the same time, 10 mg had the best effect on the studied indicators by normalizing the conduction of nerve impulses in the structures of the central nervous system, reducing excitability under stress. A toxic effect of lithium ascorbate in the studied dosages on reproductive parameters was not observed.

In the present research, we have determined that lithium ascorbate usage will vary depending on the gestation period. Specifically, a longer sow feeding time can increase the piglet survival rate and provides better litter growth in the prenatal period; the data are shown in Table 3. According to the observations of Liu et al. [31], in rat models, eicosapentaenoic acid (EPA) and docosahexaenoic acid (DHA), which are PUFAs, seem to have a defensive effect, improving SOD and GPx levels in the blood plasma of the group receiving ω-3 PUFA. It was reported that molecular mechanisms helping PUFA to induce antioxidant status stimulation is connected with Nrf2 activation and NFkB inhibition, which are two nuclear factors responsible for defense from oxidative damage and which are inflammation inducing. All these examples prove that adding sources with high antioxidant activity to the sows’ diet during gestation can be considered as effective means to increase productivity and to secure a healthy litter.

The central component of the tiol–disulfide system is glutathione. Glutathione is a tripeptide gamma glutamyl cysteinyl glycine with a free sulphhydryl group [20,21,32,33,34].

It is never found in protein hydrolysis products; therefore, glutathione is synthetized by the body for conducting specific functions. Reduced glutathione in the intracellular compartment plays a part as a main sulphhydryl bolster for maintaining cysteine residue in reduced form in all the proteins, from hematoglobulin, keeping it in a ferro form, to numerous enzymes, contained in the SH-group active center, and different vitamins, hormones and cysteamine [29,35].

Due to its chemical properties, glutathione can participate in detoxification processes by itself, reacting both with hydrogen peroxide and organic peroxide. It falls under the category of the most important thiol containing antioxidants, having antitumor and radio-protective features. Many enzymes in the active center contain sulphhydryl groups, and their oxidation leads to enzymatic activity loss [27,36,37].

An interesting feature of glutathione is that neither adding it to the food nor intramuscular and intravenous administration help. Its ability to transport through the cell membranes is poor; only reduced glutathione that has formed inside the intracellular compartment will work. Therefore, by initiating and supporting reactions leading to the preservation of reduced thiol equivalents, we can increase the body-adaptive capacity and its resistance to the impact of adverse factors [32,38,39].

Thiols occupy a special place among tissular antioxidants due to their following features: (1) extremely high reactivity to sulfhydryl groups, giving thiols the ability to oxidate with exceptionally high speed; (2) oxidation reaction reversibility of sulphhydryl groups to disulphade ones, which presumes the ability of the most energetically favorable thiol antioxidants to keep homeostasis in a cell without their biosynthesis activation; (3) ability of thiols to act as both antiradical and antiperoxile; (4) the hydrophilic nature of thiols provides their high content in the cell’s aqueous stage and gives them the possibility to be defended from oxidative damage of biologically important hydrophilic molecules, including hematoglobuli, and also due to the fact that thiols contain nonpolar groups, and are able to exhibit antioxidant activity even in the lipid phase of the cell [40,41,42,43,44,45,46,47,48]. 

Therefore, the set of indicators describing the functional condition of the glutathione reduction system was evaluated, which can be united in a consolidated set of criteria to receive conclusive and objective estimations of antioxidant and pro-oxidant processes in animal bodies [41]. These processes specifically determine animal health, fertility and the quality of products.

It is known that ineffective doses of lithium chloride (10 mg/kg) combined with an ineffective dose of ascorbic acid (0.1 mg/kg) have a synergistic effect similar to antidepressants [11].

## 5. Conclusions

Our research has shown positive effects of stress protector lithium ascorbate usage and has proven its high antioxidant activity, favorably affecting sow reproductive functions. Lithium ascorbate in dosages of 10 mg/kg per body weight given together with feed stuff shows prominent adaptogene and stress protective features in the most effective way. Lithium ascorbate improves growth gain of farrowing sows, due to better conditions of fetus formation and growth, and it secures protection from technological and spontaneous stress factors. It is possible to consider that lithium ascorbate given to sows during their reproductive cycle has a positive impact on the glutathione-reduction system, increasing livestock survivability and sow fertility indices.

## Figures and Tables

**Table 1:** Ingredients (%) and nutritional value (in 1 kg of DM) of animal feeds.

Index	Diet		
	1	2	3
	Status of Sow		
	The First 30 Days of Gestation	30–84 Days of Gestation	Last 30 Days of Gestation
Ingredients, %			
Barley	16.0	16.0	16.0
Wheat	13.0	20.0	20.0
Miller’s bran	63.5	44.5	32.5
Oats	-	-	10.0
Herbal flour	-	10.0	3.0
Sunflower mill	2.0	2.0	13.0
Meat and bone meal	2.0	2.0	2.0
Limestone meal	2.0	2.0	2.0
Salt	0.5	0.5	0.5
Premix	1.0	1.0	1.0
Nutritional value (in 1 kg of DM)			
Energy feed unit	1.16	1.08	1.16
Metabolizable energy, MJ	11.62	11.22	11.61
Crude protein, g	140.08	134.15	140.00
Digestible protein, g	105.26	101.05	104.92
Lysine, g	5.99	5.99	6.00
Threonine, g	4.09	3.94	4.10
Methionine + cysteine, g	3.60	3.38	3.61
Crude fiber, g	116.19	75.61	116.07
Salt, g	5.67	5.57	5.90
Calcium, g	8.50	8.36	8.85
Phosphorus, g	7.29	6.97	7.21
Iron, mg	80.97	76.66	80.98
Copper, mg	17.00	16.03	17.05
Zinc, mg	87.04	80.14	86.89
Manganese, mg	46.96	44.60	46.89
Cobalt, mg	1.62	1.57	1.64
Iodine, mg	0.32	0.31	0.36
Carotene, mg	11.34	10.80	11.48
Vitamins:			
Retinol (A) (thousand IU)	5.67	5.57	5.90
Ergocalciferol (D), IU	0.57	5.23	0.59
Tocopherol (E), mg	40.89	38.33	40.98
Thiamin (B_1_), mg	2.43	2.44	2.62
Riboflavin (B_2_), mg	6.88	6.62	6.89
Pantothenic acid (B_3_), mg	23.08	21.60	22.95
Choline (B_4_), g	1.13	1.08	1.15
Pantothenic acid (B_5_), mg	80.97	78.40	80.98
Cyanocobalamin (B_12_), μg	29.15	26.83	28.85

**Table 2:** Reproductive qualities of sows after administration of lithium ascorbate (M ± SD, *n* = 10).

Group	Received Piglet Heads			Birth Weight (kg)	
	Total	Alive	Stillborn	Nests	1 Head
E10	13.40 ± 1.14	13.40 ± 1.14	0	26.53 ± 1.41	1.98 ± 0.09 ^b^
E5	12.60 ± 1.52	12.60 ± 1.52	0	24.70 ± 2.14	1.96 ± 0.10
E2	11.80 ± 1.58	11.80 ± 1.52	0	22.65 ± 2.35	1.92 ± 0.09
C	10.10 ± 1.24	6.20 ± 1.58	3.70 ± 1.11	10.78 ± 2.72	1.74 ± 0.09

C—control; E—experimental groups: E10 (10 mg/kg), E5 (5 mg/kg), E2 (2 mg/kg); b—significantly compared to the control, differences significant at *p* < 0.05.

**Table 3:** Dynamics of changes in body weight of pregnant pigs after administration of lithium ascorbate (M ± Sd, *n* = 10).

Group	BW at 30 Day Gestation (kg)	BW at 60 Day Gestation (kg)	BW at 90 Day Gestation (kg)	BW at 110 Day Gestation (kg)
E10	213.40 ± 4.45	227.35 ± 4.53	247.40 ± 5.50	269.20 ± 5.07 ^a,b^
E5	215.20 ± 5.17	228.86 ± 4.95	243.80 ± 8.23	266.02 ± 9.30
E2	202.60 ± 6.69	215.20 ± 5.97	229.50 ± 8.23	252.60 ± 5.73
C	206.80 ± 7.76	217.85 ± 7.43	232.89 ± 7.83	255.00 ± 8.69

C—control; E—experimental groups: E10 (10 mg/kg), E5 (5 mg/kg), E2 (2 mg/kg). a—reliably compared to the previous period by age, differences significant at *p* < 0.05; b—significantly compared to the control, differences significant at *p* < 0.05.

**Table 4:** Activity of the glutathione reduction system in the blood against the background of lithium ascorbate (M ± SD, *n* = 10).

Day Gestation						
60 Day Gestation						
Group	SH Reduced Glutathione	SS Oxidized Glutathione	SH/SS Thiol-Disulfide Ratio	MDA Malonic Dialdehyde	SOD Activity of Superoxide Dismutase	GPx Activity of Glutathione Peroxidase
E10	1.005 ± 0.062	0.385 ± 0.041	2.63 ± 0.34	5.84 ± 0.42	1082 ± 197	2569 ± 240
E5	1.004 ± 0.126	0.420 ± 0.039	2.39 ± 0.25	6.04 ± 0.14	1023 ± 85	2525 ± 177
E2	0.938 ± 0.079	0.522 ± 0.172	1.91 ± 0.47	6.16 ± 0.75	1018 ± 94	2393 ± 128
C	0.933 ± 0.130	0.520 ± 0.129	1.87 ± 0.40	6.35 ± 1.04	1024 ± 157	2376 ± 116
110 day gestation						
E10	1.112 ± 0.058	0.432 ± 0.019 ^a,b^	2.62 ± 0.11 ^b^	4.34 ± 0.48 ^A,B^	1284 ± 55 ^a^	2803 ± 396
E5	1.089 ± 0.127	0.444 ± 0.050	2.45 ± 0.12 ^b^	5.04 ± 0.91 ^a,b^	1249 ± 239	2778 ± 236
E2	1.013 ± 0.048	0.464 ± 0.056	2.21 ± 0.25	6.68 ± 0.52	1157 ± 180	2560 ± 161
C	0.914 ± 0.185	0.524 ± 0.083	1.83 ± 0.66	7.20 ± 1.18	1140 ± 70	2346 ± 177

C—control, E—experimental groups: E10 (10 mg/kg), E5 (5 mg/kg), E2 (2 mg/kg). A, a—reliably compared to the previous period by age, B, b—significantly compared to the control. A, B—differences significant at *p* < 0.01. a, b—differences significant at *p* < 0.05. SH—reduced glutathione + cysteine, μmol/mL; SS—oxidized glutathione + cystine, μmol/mL; SH/SS—thiol-disulfide ratio; MDA—malonic dialdehyde, nmol/mL; GPx—activity of glutathione peroxidase, units; SOD—activity of superoxide dismutase, units.

## Data Availability

The data presented in this study are available on request from the corresponding author. The data are not publicly available due to privacy.
